# A network approach to understanding distance learners’ experience of stress and mental distress whilst studying

**DOI:** 10.1186/s41239-023-00397-3

**Published:** 2023-05-15

**Authors:** Rajvinder Samra, Alex Bacadini França, Mathijs F. G. Lucassen, Philippa Waterhouse

**Affiliations:** 1 School of Health, Wellbeing and Social Care, The Open University, Horlock H020, Walton Hall, Milton Keynes, MK7 6AA UK; 2 Laboratory of Human Development and Cognition, Federal University of São Carlos, São Paulo, Brazil

**Keywords:** Stress, Mental distress, Network analysis, Distance education, Students

## Abstract

Research has shown that learners’ stress and mental distress are linked to poorer academic outcomes. A better understanding of stress and mental distress experiences during study could foster more nuanced course and intervention design which additionally teaches learners how to navigate through to protect their academic performance. The current study draws on data collected via validated self-reported questionnaires completed by final year undergraduate students (n = 318) at a large distance education university. We examined how common features of stress, depression and anxiety link to each other using a network analysis of reported symptoms. The results included findings demonstrating the symptoms with the greatest relative importance to the network. Specifically, these included the stress symptom ‘I found it difficult to relax’ and the depression symptom ‘I was unable to become enthusiastic about anything’. The findings could help institutions design interventions that directly correspond to common features of students’ stress and distress experiences.

## Introduction

Internationally, the higher education sector is dealing with concerning rates of student mental ill-health and distress. Recently, the World Health Organization’s (WHO) World Mental Health International College Student (WMH-ICS) project has led a series of surveys to estimate the extent of the problem across a number of countries, including Australia, Belgium, Germany, Mexico, Northern Ireland, South Africa, Spain, and the United States (Auerbach et al., 2018; Husky et al., 2022). Auberbach et al. (2018) reported that approximately 31% of first year students, from a sample of 13,984 respondents in eight countries across 19 colleges, screened positive for at least one mental health disorder (such as major depression, generalized anxiety disorder or panic disorder) in the twelve months preceding the survey. Alongside the high rates of student mental ill-health, evidence indicates greater usage of campus-based mental health services across countries (Flatt, 2013; Lipson et al, 2019; Universities Ontario, 2017). However, as university-based mental health services struggle to match increased demand, there are key questions around how a student’s mental health needs may affect their study experiences.

Students’ mental health significantly impacts upon their studies. For instance, Eisenberg et al. (2009) conducted a longitudinal study of college students in the USA and found that depression scores at baseline predicted a lower Grade Point Average (GPA) and greater probability of dropping out of their studies at a three-year follow-up point. Results indicated that co-occurring depression and anxiety were especially impairing to a student’s GPA. Additional studies corroborate a statistical relationship between mental distress and poorer academic outcomes (Auerbach et al., 2016, 2018; Ishii et al., 2018). For example, Ishii et al.’s (2018) retrospective analysis of the medical records of 203 undergraduate students showed that students receiving treatment for a mental illness had higher drop-out rates than matched controls. Factors that correlated with the significantly higher dropout rates for students receiving treatment included social withdrawal, temporary leave of absence and extended enrollments (Ishii et al., 2018). Furthermore, Bruffaerts et al.’s (2018) analysis of WMH-ICS’s data from Belgium found that students with internalizing symptoms (e.g., depression and anxiety, post-traumatic stress and suicidal ideation) or externalizing symptoms (e.g., inattentiveness, hyperactivity, impulsivity and conduct disorder) had a lower GPA after controlling for confounders. Additionally, the authors reported that internalizing mental health problems showed more consistent patterns with lower academic functioning, than externalizing experiences, and this finding highlights the need to attend to the key features of the student’s distress experiences. These studies indicate that some aspects of students’ study experiences can be made more visible at the institutional level, for educators and researchers, should we seek to better understand students’ experiences of mental distress. Such an approach requires more fine-grained exploration that transcends traditional measurement ‘levels’ or cutoffs and focuses more on patterns and features of students’ stress and mental distress.

In addition to the mental ill-health or mental distress that students often face, there are questions around the role that general life stress and academic stress may play in a student’s academic journey and on their wellbeing. The experience of stress is a commonplace and impactful aspect of a student’s life. In a sample of 20,842 students across nine countries, the majority of student respondents (94%) reported at least some form of stress (e.g., financial, health, relationships with family, work and peers, and the health of loved ones) (Karyotaki et al., 2020). In the USA, the third version of the National College Health Assessment survey reported that approaching half (44%) of the 53,720 undergraduate student respondents stated that stress negatively impacted on their academic performance (American College Health Association, 2022). More students identified the negative impacts on their academic performance from stress (i.e., 44%) than from the impacts of anxiety (37%), depression (28%) and sleep difficulties (26%). This study was nationally representative and the largest data set on college students’ health and wellbeing in the USA (American College Health Association, 2022). The results reinforce that stress is a major factor that negatively affects a student’s academic performance. Moreover, Richardson et al.’s (2012) earlier systematic review and meta-analysis of 2452 datasets pertaining to psychological correlates of university students' academic performance from 1997 to 2010 identified general stress and stress related to academic work as significantly negatively correlated to GPA. Although the correlations were small, they are still important to note given the scale of the review and meta-analysis. Similarly, Amirkhan and Kofman’s (2018) study on stress and academic performance demonstrated that stress overload, in a sample of over 500 first-year undergraduate students in the USA during their first semester, predicted their end-of-year GPA better than most traditional indicators (such as hours engaged in paid work, units/courses enrolled, parental education, parental income, and gender). Amirkhan and Kofman (2018) recommend that measures of stress overload may represent a useful “red flag” (p. 297) to predict poor grades and subsequent attrition. Thus further supporting the idea that understanding stress, how it manifests and also how it affects students’ academic performance is timely concern and an important opportunity.

Given the findings demonstrating that stress and mental distress show clear links to reduced academic performance (e.g., Ishii et al., 2018; Richardson et al., 2012), this invites the question of what are the common features or symptoms in students? For instance, how do symptoms relate to each other to create patterns of mental distress which could go on to impair learning or performance? Therefore, there is an opportunity for educational researchers to more deeply explore and better understand the student experience in relation to stress and mental distress. This in turn would help inform the response that universities could offer to prevent reduced academic performance and mitigate against the higher risk of course drop-out. There is increased emphasis, for example in the United Kingdom (UK) student mental health charter, that interventions need to go beyond the provision of conventional mental health services and should extend to include those related to educational practices and culture more generally (Hughes & Spanner, 2019).

The current paper examines in greater detail the symptoms of stress and mental distress in a sample of distance education students. We define mental distress as a “state of emotional suffering characterized by symptoms of depression and anxiety” (Drapeau et al., 2012, p. 105). Our use of the term ‘stress’ is inclusive of general life stress or academic stress in line with prior work (e.g., Richardson et al., 2012). Participating students did not have to self-disclose a mental illness in order to take part. This inclusive approach to participation is important, as higher education providers may not be able to rely upon flags or indicators relying on of self-disclosure, as certain students with a diagnosed mental disorder may never formally disclose this to an educational provider. For example, Brown et al. (2018) found that the perceived stress and public stigma surrounding mental ill-health was associated with lower levels of disclosure among college students in the USA with a mental illness, particularly among men in their sample. Furthermore, not all students with symptoms of depression and anxiety may identify them as such or have a formal diagnosis of a specific mental health disorder. Hence, there is a need to understand how all students experience mental distress and stress more generally during their studies, irrespective of whether or not they have disclosed a mental illness to their higher education provider.

## Literature review

### Managing stress and distress during academic study

The role of affective skills and emotional regulation skills are described by the research literature as useful for managing stress and mental distress (e.g., Bryant & Malone, 2015; Houghton et al., 2011; Pepping et al., 2013). However, there is also a growing literature on the role of improving one’s cognitive skills and self-confidence in using these skills, to mitigate against mental health disorders through developing one’s metacognitive processes (e.g., Chinaveh et al., 2010; Wells & Cartwright-Hatton, 2004). Metacognition refers to the evaluation, control, and monitoring of one’s thought processes (Flavell, 1979). Metacognition can also relate to an awareness and monitoring of one’s learning processes (Celik, 2022). Having greater metacognitive skills predicts higher academic success in undergraduate students (Celik, 2022), with metacognitive skills also thought to play a role in the development and maintenance of mental health disorders (Wells & Matthews, 1996). Importantly, Flavell (1979) states that metacognitive experiences during learning include affective (emotional) experiences, because these have important effects on cognitive tasks and task completion and are part of motivation. For example, feelings of failure or inadequacy can influence how one determines and executes strategies for goal completion. Thus, stress, anxiety, and depression may be seen to exert a negative impact on a student’s ability to concentrate, learn and maximize their educational opportunities by interfering with their own metacognitive and self-regulatory capacities.

We might consider that the main symptoms that activate stress and mental distress among students could interfere with their learning. As a result, it would be useful to better understand the network model of these symptoms, not in the context of levels of distress but in the context of what is experienced and how different feelings and symptoms commonly link to each other. An examination of psychological stress and distress in students can therefore usefully inform better metacognitive strategies around how students predict, manage, and develop skills about how their emotions might impact upon their learning. For example, understanding students’ mental distress and stress symptoms may engender teaching and educational content that promotes improved cognitive skills, such as effective problem-solving, when a student becomes aware of their specific mental stress and distress, akin to a ‘signature’ pattern of symptoms, which serve as an early warning signal to oneself or others. Brougham et al. (2009) posits that higher education is an appropriate place for students to develop the skills that they may need to avoid dysfunctional stress or burnout in their future lives. As such, institutional understanding of their students’ experiences of mental stress and distress can inform the future development of strategies that help students to develop their self-regulatory skills, at university and beyond.

### Exploring networks of stress and distress

A network analysis approach is well-suited to examine how stress and mental distress symptoms link to and activate each other. Network analysis is an innovative approach that is gaining popularity in psychometric research and importantly, it assumes the dynamic interaction of different symptoms of mental distress without assuming an underlying latent construct, such as in latent models of depression or generalized anxiety disorder (e.g., Borsboom et al., 2013). In network analysis, symptoms (i.e., ‘nodes’) are examined for their role in the network, such as whether they are central to the network and whether they tend to have an important role in terms of activating other symptoms of distress (e.g., act as ‘bridges’ to other symptom communities). Therefore, a network approach as applied to stress and mental distress in students could elucidate how symptoms of stress, anxiety and depression activate each other. The focus is not on whether a student has or does not have a mental health disorder, but about the common experiences of these symptoms and how they relate to each other.

Network analysis has been rarely applied to study student mental distress. A notable exception is Makhubela’s (2021) study in South Africa which explored symptoms of depression and anxiety in a sample of undergraduate students, using measures suitable for a non-clinical population. The analysis revealed that the symptoms of anhedonia (i.e., the inability to feel pleasure), hopelessness, worthlessness, self-blame, and loneliness had the strongest connections to other symptoms in the network. Makhubela (2021) concluded these represent the most important aspects to target via interventions to support undergraduate students. Similarly, Frewen et al. (2013) used network analysis to explore connections between symptoms of anxiety, posttraumatic stress, and depression in a sample of Canadian undergraduate students, identifying anxious worrying, depressed mood and remembering or being reminded of traumatic events as central symptoms in the network. More recently, Bai et al. (2021) drew on data collected from nursing students in China and found that symptoms of depressed mood, nervousness and anhedonia linked anxiety and depression communities by acting as bridges. Bridges may link symptoms from one community (e.g., anxiety) to another (e.g., depression) and may represent useful points of intervention for educational institutions.

A limitation of Makhubela’s (2021), Frewen et al.’s (2013) and Bai et al.’s (2021) research of mental distress in university students is the absence of the consideration of stress in their network analyses. Past research has identified the role of stress in reduced academic performance (e.g., Richardson et al.,’s 2012 systematic review and meta-analysis). More recently, McIntyre et al.’s (2018) research among 1135 undergraduate students in the UK found specific forms of stress (including some types of discrimination, loneliness, and academic stress) to be associated with higher severity level of anxiety and depression, as measured by validated self-reported questionnaires. Therefore, the inclusion of symptoms of stress in activating depression and anxiety symptoms represents an important gap in the knowledge base which is addressed in the present study.

To the best of our knowledge, to date, there has not been a network analysis of mental distress amongst distance education students. The lack of knowledge about the experience of stress and distress in distance education students also represents an important and significant gap in the knowledge base. There are additional key reasons why findings from a network analyses of general university student populations may not be applicable to distance education students. Distance education students, typically, experience different or additional stressors compared to students studying face-to-face at a university. Whilst distance education students are likely to encounter some of the same stressors as their campus-based counterparts, such as the academic demands associated with studying towards a degree or qualification, research indicates that the very nature of studying at a distance can create extra challenges. These include distance students’ frequently experiencing loneliness and isolation (Markova et al., 2017) alongside having to adjust to, and skillfully navigate, complex virtual learning environments to access teaching (Wozniak & McEldowney, 2015). The flexibility of distance education allows students to combine their studies more readily alongside their pre-existing commitments, and this is often cited as the predominant reason for adults choosing this mode of study (Ashton & Elliot, 2007). As a result, distance education students routinely combine their studies alongside work and family responsibilities, and for some, this can create additional sources of stress.

Given the global growth in distance education since 2010 (Qayyum & Zawacki-Richter, 2019) the needs of students studying in this way warrants further attention. This coupled with the fact that students who are remote from physical campuses cannot easily access the counselling and support services that have been traditionally offered in the case of students at face-to-face institutions. Innovations in higher education mean many universities are reconsidering their teaching approaches so that they become ‘blended’ (Naidu, 2021), and given its popularity some are likely to expand their distance education offerings in the future, especially if there are sufficient financial and pedagogical motivations. Therefore, it becomes pertinent for a range of higher education providers to better understand how stress and distress are modelled in students who study at a distance, in order to better support their students’ academic performance. The current study examines how stress as well as depressive and anxiety symptoms relate to each other in a sample of distance education university students. This work is a secondary analysis of data on mental distress collected via a validated self-reported questionnaire completed by undergraduate students at a large distance education university in the UK. The original study was designed to measure levels of student distress and its relationship to student characteristics using traditional regression-based analyses (see Waterhouse et al., 2020). However, such analyses reveal very little of the nature of the experiences of student distress and this knowledge gap represents an important opportunity to re-examine the data from a network perspective. A knowledge gap exists around how symptoms of distress are linked to each other in the distance education student population, and what this might mean for practical support for students. This study will contribute to the evidence-base and inform the design of interventions by identifying how symptoms of stress, depression, and anxiety may activate each other.

Hence, the research objectives of the present study were:

- To use network estimation to explore the network structure of stress, anxiety and depression symptoms of distance education students;
- To investigate which symptoms of depression, anxiety or stress are the most important to the network as a whole;
- To determine which symptoms may link the different communities (e.g., depression, anxiety, and stress).

## Materials and methods

### Institutional context

The data used in this study was collected from students studying modules in the School of Health, Wellbeing and Social Care and the School of Education, Childhood, Youth and Sports at The Open University. With currently over 200,000 students enrolled, The Open University takes a multi-disciplinary approach to course development where academics, educational technologists and media specialists contribute to the design of learning guides (The Open University, 2022). These learning guides contain computer mediated activities and are hosted on a virtual learning environment. Students are also supported via tutorials run using online conferencing and course discussion forums.

### Participants and procedures

A sample of 1436 students enrolled in final/third year health and social care or education modules were invited, via email, to take part in an online questionnaire. The present study was situated within a larger project that aimed to explore the relationship between students’ work and family responsibilities and mental distress. The full survey collected quantitative data related to students’ work and family roles (particularly their partnership, parenting, and caregiver status), the experience of conflict and facilitation between work and family roles with study, and a validated measure of mental distress (See Waterhouse et al., 2020; Samra et al., 2021). Qualitative data was also collected via four open-ended questions that asked the strategies that respondents used to combine studying with their work and/or family roles; perceived effectiveness of university support; and recommendations for the university. The questionnaire, as well as the information sheet and consent form, were pilot tested with five individuals, including current students at the university and they were refined based on feedback. The study received ethical approval from The Open University Human Research Ethics Committee (HREC/3165/Waterhouse). The survey was open for three weeks in April 2019.

### Measures

Symptoms of mental distress were measured using the Depression, Anxiety and Stress Scale short-form (DASS-21, Lovibond & Lovibond, 1995). This is a standardized self-reported assessment of mental distress designed for the general population and has been used frequently to research the mental distress of students in higher education (e.g., Bayram & Bilgel, 2008; Jones et al., 2019; Larcombe et al., 2014; Osman et al., 2012). The DASS-21 consists of three sub-scales (i.e., a depression, anxiety, and stress scale) that each contain seven items with a four-point scale which describes how often respondents experienced symptoms in the week preceding the survey (ranging from 0 did not apply to me at all to 3 applied to me very much or most of the time). The depression scale assesses dysphoria (a persistent state of unhappiness and dissatisfaction), hopelessness, devaluation of life, self-deprecation, lack of interest/involvement, anhedonia, and inertia. The anxiety scale assesses autonomic arousal, skeletal muscle effects, situational anxiety, and subjective experience of anxious affect. The stress scale assesses difficulties relaxing, nervous arousal, and being easily agitated, irritable and impatient.

### Statistical methods

The network analysis was conducted in two steps: network estimation and network description.

#### Network estimation

The relationship among specific depression, anxiety, and stress items was explored using a Gaussian Graphical Model (i.e., a partial correlation network). We estimated the network applying the nonparanormal transformation and then computed correlations (Isvoranu & Epskamp, 2021) and the Least Absolute Shrinkage and Selection Operator (LASSO) regularization technique to control for spurious interactions. The LASSO regularization parameter was selected through the Extended Bayesian Information Criterion (EBIC), with the hyperparameter gamma set to the default value of 0.50 for model selection (Epskamp et al., 2018). In the resulting network, nodes represented questionnaire items and the relationships between them were represented by edges, the weight of the edges representing the strength of association between two variables after controlling for the others. Items were previously allocated in their respective dimensions as described in the DASS-21 scale version. All statistical analyses were performed using R-packages: qgraph 1.6.9, bootnet 1.4.3.

#### Network description and node characteristics

We evaluated the relative importance of nodes using strength centrality, which is the sum of all the weights connected to a given node, in absolute value. This index tells how much a node is well connected to all other nodes in the network. Moreover, the bridge strength (i.e., the sum of the absolute value of all edges that exist between a node A and all nodes that are not in the same community as node A) and bridge betweenness (i.e. the number of times a node B lies on the shortest path between nodes A and C, where nodes A and C come from different communities) (e.g., depression-anxiety, depression-stress, anxiety-stress) were checked. We also evaluated the predictability index. Predictability is defined as the variance in a node that is explained by all other nodes in the network. Predictability gives us a proportion of how much a node’s variance is explained by other nodes in the network. In the network plot, the ring-shaped pie charts represent predictability (Haslbeck & Waldorp, 2018). High predictability indicates the extent that the network is determined by its own strong interactions with other nodes, whereas low predictability indicates that it is determined by factors not included in the network (Haslbeck & Waldorp, 2018).

The robustness of strength, bridge strength, and predictability was examined by calculating indices of accuracy and stability of the results. The stability of node properties was estimated using a case dropping bootstrap procedure. We focused on the correlation stability coefficient (CS-coefficient), the proportion of the sample that could be dropped while still retaining with 95% probability a correlation between the index computed on the full sample and the index computed on the reduced sample: A value below 0.25 indicates insufficient stability, whereas a value larger than 0.50 is preferred (Epskamp et al., 2018). Strength (CS = 0.65), Bridge strength (CS = 0.45) and Predictability (CS = 0.75) were shown to be stable and the results presented. Bridge closeness (CS = 0.20) and bridge betweenness (CS = 0.05) were not found to be stable. It is important to say that closeness and betweenness generally do not show stability and can be problematic measures, since they treat association as distances (Borsboom et al., 2021; Epskamp et al., 2018; Hallquist et al., 2021). The results for bridge betweenness and closeness therefore are not reported.

## Results

### Sample description

The overall response rate for the questionnaire was 24.2% (n = 348). Those with missing data were excluded from further analyses which resulted in 318 students in the final sample. Table 1 presents the descriptive statistics for the sample. The highest level of educational attainment at time of registration to the university varied in the sample. Less than a quarter had ‘less than A-Levels or equivalent’, 41% had A-levels or equivalent and 36% had some form of higher education qualification. By way of context, A-levels are a UK (except for Scotland) subject-based qualification for those aged 16 years and older. Equivalent qualifications include Scottish Highers and the International Baccalaureate. Eighty-eight percent of the sample were female and 85% reported their ethnicity to be white. Whilst there was an element of non-response bias by gender (i.e., females were more likely to respond to the survey), the predominance of females to some degree reflects the demographics of students on the modules, from which the sample was selected. The age profile of the sample was diverse, as reflective of distance education students generally, and included 41% of the sample being over the age of 40 years. The vast majority of students (89%) had engaged in some form of paid employment in the month preceding the survey, and just over half lived in a household with children aged 18 years or younger.

**Table 1:** Socio-demographic characteristics of the sample (n = 318)

Variable	n	%
*Worked in past month*		
Yes	284	89.31
No	34	10.69
*Partnership status: currently married, in civil partnership or cohabiting*		
Yes	214	67.30
No	104	32.70
*Parenting status: children aged 18 years and younger living in same household*		
Yes	167	52.52
No	151	47.48
*Caregiver status: unpaid carer*		
No	215	67.61
Yes, 1–19 h a week	63	19.81
Yes, 20–49 h a week	16	5.03
Yes, 50 + hrs a week	24	7.55
*Sex*		
Female	279	87.74
Male	39	12.26
*Ethnicity*		
White	270	84.91
Non white	48	15.09
*Highest educational attainment at time of registration to university*		
Less than A levels	75	23.58
A levels or equivalent	130	40.88
Higher education attainment	113	35.53
*Age (years)*		
19–24	33	10.38
25–29	48	15.09
30–34	52	16.35
35–39	54	16.98
40 +	131	41.19

### Network estimation and node characteristics

**Fig. 1:**
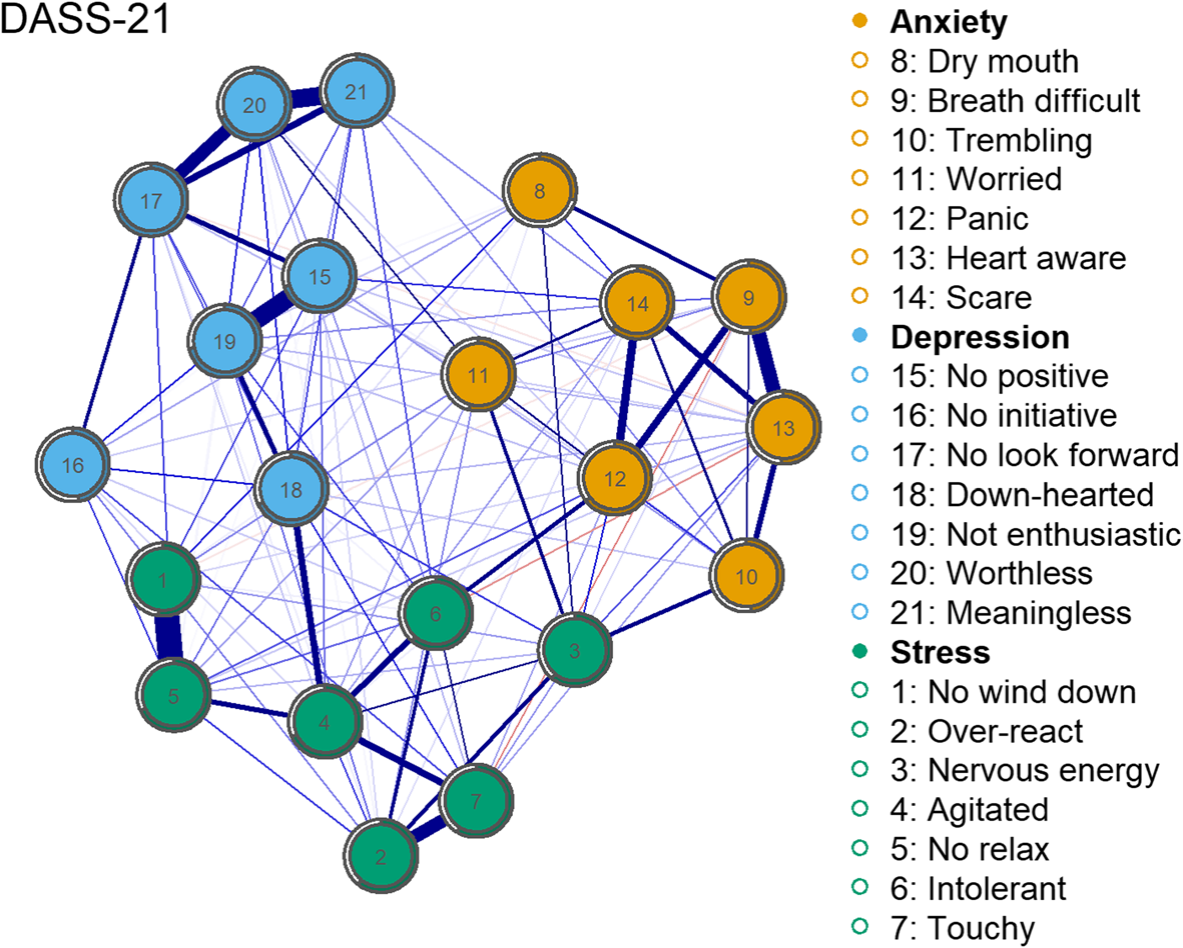
The DASS-21 network with community analysis (N = 318). Ring-shaped pie charts represent predictability. The nodes are arranged in order according to the legend. Yellow nodes represent anxiety symptoms, blue nodes represent depression symptoms and green nodes represent features of stress. The size and density of edges between nodes represent the strength of connectivity

Figure 1 presents the network for the entire sample. The network represents the symptoms of anxiety, depression and stress measured by Depression Anxiety Stress Scales (DASS-21). The nodes (i.e. the items in the DASS-21) were organized by subscale: Anxiety, Depression and Stress. The items within each community are outlined in the Appendix.

As depicted in Fig. 1, the network consisted of solely positive edges, with many strong edges between nodes. Strong edges were presented within clusters rather than connecting with other communities. With respect to anxiety symptoms, strong edges were presented between nodes denoting that respondents ‘experienced breathing difficulty’ (#9), felt ‘close to panic’ (#12), felt a ‘sense of heart rate increase, heart missing a beat’ (#13) and felt ‘scared without any good reason’ (#14). Being ‘aware of dryness of my mouth’ (#8) and being ‘worried about situations in which I might panic and make a fool of myself’ (#11) were both poorly connected within the anxiety community. Within the depression community, strong edges were presented between the nodes ‘I felt that I had nothing to look forward to’ (#17), ‘I felt I wasn’t worth much as a person’ (#20) and ‘I felt that life was meaningless’ (#21). The nodes ‘I couldn’t seem to experience any positive feeling at all’ (#15) and ‘I was unable to become enthusiastic about anything’ (#19) emerged as particularly strongly connected. On the other hand, ‘I found it difficult to work up the initiative to do things’ (#16) and ‘I felt down-hearted and blue’ (#18) were both poorly connected within the depression community. The nodes in the stress community that showed a strong association were ‘I found it hard to wind down’ (#1) and ‘I found it difficult to relax’ (#5). Of the remaining nodes, ‘I tended to over-react to situations’ (#2), ‘I found myself getting agitated’ (#4), ‘I was intolerant of anything that kept me getting on with what I was doing’ (#6), and ‘I felt I was rather touchy’ (#7) emerged intrinsically connected to each other.

### Strength and bridge strength

**Fig. 2:**
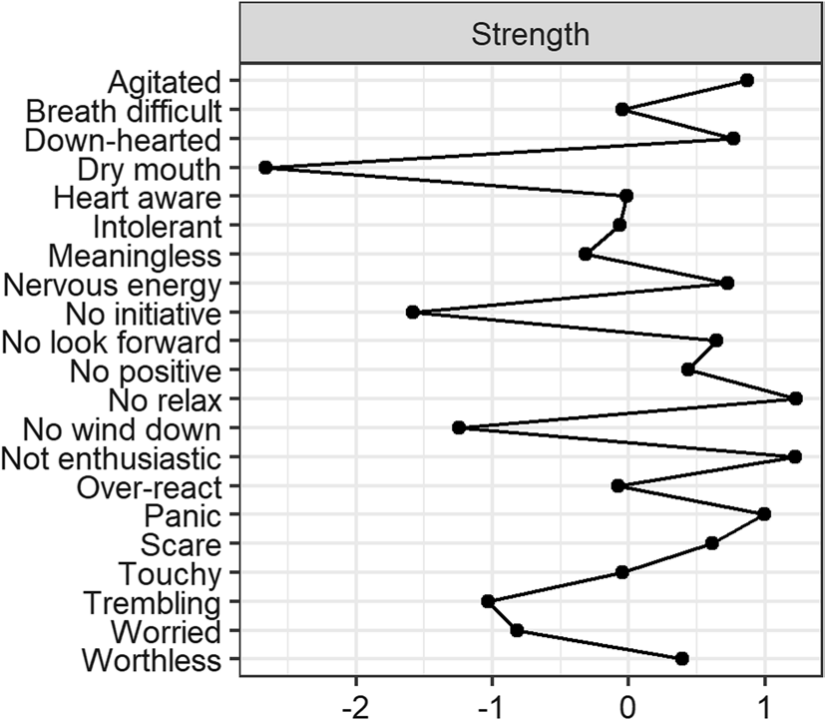
Strength centrality shown as standardized z-scores

Figure 2 shows the standardized strength scores for each node. Overall, the symptoms with the highest standardized strength centrality were finding it difficult to relax (#5) from the stress community, feeling unable to become enthusiastic about anything (#19) from the depression community, and feeling close to panic (#12) from the anxiety community.

Communities may bridge to each other if the symptoms of one disorder activate symptoms in another. The nodes with the largest standardized bridge strength scores, and those that most strongly acted as bridge symptoms across the network were ‘I felt that I was using a lot of nervous energy’ (#3, stress), ‘I felt down-hearted and blue’ (#18, depression), and ‘I was worried about situations in which I might panic and make a fool of myself’ (#11, anxiety).

### Network predictability

Predictability scores are shown as ring-shaped pie charts in Fig. 1. Mean predictability across all nodes was 60% (i.e., 60% of the variance of a node that is not predicted by the intercept model is explained by its neighbors) in the entire sample network. The nodes with the highest predictability statistics and thus important to the entire network sample all came from the depression community. Specifically, these included ‘I was unable to become enthusiastic about anything’ (72% predictability, #19, depression), ‘I couldn’t seem to experience any positive feeling at all’ (70% predictability, #15, depression), ‘I felt that I had nothing to look forward to’ (69% predictability, #17, depression) and ‘I felt I wasn’t worth much as a person’ (69% predictability, #20, depression).

## Discussion

The literature indicates the importance of stress and mental distress on academic outcomes (e.g., Auerbach et al., 2016; Ishii et al., 2018). However, despite research indicating the considerable prevalence of the experience of stress and mental distress by university students (e.g., Auerbach et al., 2018; Jenkin et al., 2021; Jones et al., 2019), knowledge gaps exist around the particular signs and symptoms that students’ experience during their learning journey. This study used an innovative method and a validated measure of depression, anxiety, and stress to explore the network structure of mental distress in a non-clinical sample of undergraduate online distance education students. To the authors’ knowledge, it is the first network analysis of mental distress in a distance education student sample. We used the DASS-21 measure, a validated measure, which is inclusive of stress symptoms and so this work usefully adds to the recent work that has explored networks of distress, namely depression and anxiety (e.g. Bai et al., 2021; Makhubela, 2021). Our results may have relevance to other settings in which students are not proximally based within a physical campus culture and climate. This work helps illustrate how students’ affective experiences are linked to each other to contribute to overall mental stress and distress during their study journey. The results and potential implications for the design of practical interventions are discussed below.

The strength centrality metrics conducted in the current study provide information about the relative importance of a symptom in the network (Opsahl et al., 2010) and may provide important clues to clinical and practical research by suggesting potential targets for interventions in order to ‘deactivate’ symptoms using network connections (Rodebaugh et al., 2018). In this study the symptoms with the highest standardized strength centrality scores were ‘I found it difficult to relax’ and ‘I was unable to become enthusiastic about anything’. This finding has partial overlap with Bai et al.'s (2021) network analysis of mental distress symptoms among Chinese nursing students that found trouble relaxing to constitute a central symptom alongside symptoms of irritability and uncontrollable worry, the latter two of which are less important in our network. From a higher education perspective, ours and Bai et al.’s (2021) results indicates that interventions that promote better relaxation during the most stressful times of study may be particularly beneficial to design into courses at particular timepoints (e.g. in preparation for important assignments and exams). The centrality of the symptom ‘I was unable to become enthusiastic about anything’ in our study indicates that practitioners may want to consider ways to encourage students to be enthusiastic in their course, or subject by the thoughtful use of activity design during latter and more difficult stages of study. Additionally, reminding students of the wider personal meaning and purpose of their study choices and course selection could be used as a potential intervention to address this central feature of the feelings of depression students might experience during the latter stages of their study. Interventions focusing on alleviating specific central symptoms should, theoretically, promote a beneficial cascade effect, whereby improvement in one symptom triggers improvements in other symptoms and impacts on the other behaviors, thoughts, and emotions related to the core symptom (Boschloo et al., 2016).

The consideration of bridge strength allowed us to explore which symptoms in the network link the different communities (i.e., the depression, anxiety and stress). ‘I felt that I was using a lot of nervous energy’ (stress community) was the symptom with the highest standardized bridge strength score. Of the depression symptoms, ‘I felt down-hearted and blue’ was the most important in activating symptoms in other communities, whilst ‘I worry about situations in which I may panic and make a fool of myself’ was the symptom that had the highest standardized bridge strength score in the anxiety community. Similarly, this symptom about worrying about panic and embarrassment most strongly connected the anxiety community to the depression community in Van den Bergh et al.’s (2021) network analysis of the DASS-21 in a large international sample of adults. The authors argue to people who worry about panicking in future situations are more likely to also be ruminative about past situations, which is characteristic of depression, and explains why and how these particular anxiety symptoms bridge to feelings associated with depression. Van den Bergh et al. (2021) also found 'I felt down hearted and blue’ and ‘I felt that I was using a lot of nervous energy’ to be potential bridges between communities. Research conducted in large-scale cross-national representative surveys of general adult populations (Kessler et al., 2015), as well as more specific student populations (Auerbach et al., 2018), have demonstrated considerable comorbidity of mental health disorders. Given these findings, the use of network approach to identify potential points of connection between symptom communities is a considerable strength and suggests potential cost-effective points for intervention in light of common comorbid conditions such as depression and anxiety.

Feelings of hopelessness, pessimism and worthlessness (i.e., symptoms of depression) stood out with the highest predictability scores which indicates that to a large degree they are predicted within the network (by their neighboring nodes) rather than caused by external or outside factors (Haslbeck & Waldorp, 2018). For symptoms that are high in predictability, taking a holistic approach and designing interventions that target neighboring nodes/symptoms may be a useful point of entry for intervention to reduce their levels. For example, the feeling of not having anything to look forward to is a neighboring node to students’ feelings of worthlessness, which can help us better understand students’ needs for perhaps social events or positive connection to boost morale during these periods of feeling low. Bewick et al. (2010) longitudinal analysis of depressive scores of undergraduate students over three years of study showed a consistent steady increase over each semester reaching highest levels during the final year. This is perhaps counterintuitive to notions of the institutional perspective of how positively transforming an undergraduate degree is assumed to be. It is also counterintuitive to the idea that students will feel increasingly excited to near graduation at the thought of course completion and graduation. Our results and Bewick et al.’s (2010) raise important questions about how to encourage and support meaning and worth for students who have been subjected to evaluation for many years. Given the increasingly competitive job market, Bewick et al. (2010) suggest interventions that support and prepare students for the transition from study to employment are particularly important at the final stages of a study journey.

In line with Flavell’s (1979) acknowledgment of how affective experiences form part of the metacognitive processes that underpin learning, we might use these findings to inform course design to predict greater likelihood of particular thoughts and feelings at certain time points. For example, we can recognize that latter-stage students may need to be encouraged to reflect positively on their own improvements during their overall journey. This should in turn reinforce hope and worth at this final stage when their mood may be lower. This may also lessen the potential destructive influence for some students who see the looming threat of a final degree grade and finding employment which might affect their self-confidence, but also interfere with their learning in the latter stage of their course.

### Strengths and limitations

An important strength of the present work is the deliberate inclusion of stress as located proximally to students’ distress experience in line with previous work which highlights that students perceive a key role for stress in impairing their academic performance, along with depression, anxiety and sleep problems (American College Health Association, 2022). In our results, symptoms located in the stress community were identified as of particular importance to the overall network based on their strong edges. We also found that symptoms in the depression community was tightly interconnected with high predictability scores, suggesting there are important knock-on activation effects once a depression symptom becomes activated. Our study builds on recent past work using network analyses in undergraduate samples which has focused on mental distress by measuring depression and anxiety (Bai et al., 2021; Makhubela, 2021), but develops this field with the inclusion of how feelings of stress manifest and relate to symptoms of anxiety and depression. Our results demonstrate a tightly connected DASS-21 network, which supports the notion that stress is an important psychological construct to include and measure in conjunction with mental distress to more fully understand students’ distress-related emotional experiences whilst studying and learning.

The current study has some limitations. The present work is correlational and conclusions about causality cannot be drawn. Van den Bergh et al. (2021) highlight that variables not included in network analysis of symptoms of distress, such as biological predisposition and external stressors, may change the strength of edges. Network analysis is a population level analysis and cannot explore variations that may exist at the individual level. Our sample was also relatively small and mostly comprised of women who are white-identified, which is in line with the course registrations on the modules sampled, but does not reflect other and more diverse contexts. Therefore, we were unable to conduct sub-sample analysis to explore possible differences in the network structure by sociodemographic characteristics. Different samples have shown inconsistent findings with regards to gender differences (e.g., Bai et al., 2021; Makhubela, 2021; Van den Bergh et al., 2021). Further work comparing gender differences in a distance education student sample is needed to explore whether networks differ, for instance between men and women and non-binary students. Another limitation of the study was that the data was collected prior to the COVID-19 pandemic and lockdowns. Whilst this is a limitation, it also presents an opportunity for future work to draw comparisons on DASS-21 network analyses before, during and after the pandemic. As a limitation of network analyses, the extent that the network identified represents other samples is unknown and so caution must be taken in generalizing results to other populations.

## Conclusions and recommendations

Our study reported on the structure of symptoms of stress and mental distress in a sample of distance education students. This study can inform work focused on students’ distress-related emotional experiences, whilst in the latter stages of undergraduate study at a distance and without proximity to a physical campus. Our results demonstrated a very dense network structure of mental distress symptoms, with several bridging edges in which particular symptoms linked the different communities (i.e., stress, anxiety and depression). A tightly interconnected network increases the probability of symptoms activating other symptoms and consequently, putting an individual at risk of developing symptoms that can come to characterize or develop into a second mental health disorder. This approach to understanding student mental distress that considers the dynamic interaction of different symptoms of distress extends knowledge because it is a departure from the research tradition of using sum scores or composites to identify the level and extent of depression, anxiety or stress in distance education students (e.g., Jones et al., 2019; Waterhouse et al., 2020).

In terms of interventions, applying a metacognitive stance, higher education institutions could design interventions to promote better planning, monitoring, and evaluation of students' emotional and motivational strategies. For example, teaching students about how to reflect on stress management and relaxation strategies that they have (successfully and unsuccessfully) employed in the past, in order to build their self-awareness and confidence in managing mental stress and distress associated with their study experiences. Encouraging students on how to monitor their stress and distress levels using central symptoms or bridging symptoms could also promote students to identify times when they need to take action or seek institutional assistance in managing distress and stress to support their academic experience and performance. From a course design perspective, cognitive activities (such as planning and problem-solving) can be designed to facilitate students self-learning and self-discovery about their emotional and motivational states whilst they study. Building awareness and strategies for action may improve students’ confidence in their ability to cope with burgeoning stress during the latter stages of study and subsequently equip students during their studies and in any future careers in which they may face increased job stress. Ultimately this trajectory is one of building self-awareness regarding emotional and motivational management in order to promote academic performance. This is in line with past research which demonstrates that academic self-efficacy and stress are negatively correlated (Ye et al., 2018; Zajacova et al, 2005).

Furthermore, the present work is in line with research that acknowledges the role of affective aspects of experiences when pursuing cognitive tasks such as studying (e.g., Flavell, 1979). If institutions can conceptualize emotional and motivational elements as part of the academic experience and seek to design courses that are mindful of changes in student stress and distress, higher education could be a place where students learn the life and emotional management skills to support their cognitive performance as they study. There is an opportunity to develop institutional course design to include emotional and self-regulatory learning in a way that could protect students from impaired academic performance due to stress and distress as well as better protect against career and job burnout in their post-university life (Brougham et al., 2009; Deasy et al., 2014).

## Data Availability

The data that supported this study are available upon request from the corresponding author.

## Appendix

DASS-21 items for depression, anxiety and stress subscales.

**Table:**

Item number	Community	Item
1	Stress	I found it hard to wind down
2		I tended to over-react to situations
3		I felt that I was using a lot of nervous energy
4		I found myself getting agitated
5		I found it difficult to relax
6		I was intolerant of anything that kept me from getting on with what I was doing
7		I felt that I was rather touchy
8	Anxiety	I was aware of dryness of my mouth
9		I experienced breathing difficulty (e.g. excessively rapid breathing, breathlessness in the absence of physical exertion)
10		I experienced trembling (e.g. in the hands)
11		I was worried about situations in which I might panic and make a fool of myself
12		I felt I was close to panic
13		I was aware of the action of my heart in the absence of physical exertion (e.g. sense of heart rate increase, heart missing a beat)
14		I felt scared without any good reason
15	Depression	I couldn’t seem to experience any positive feeling at all
16		I found it difficult to work up the initiative to do things
17		I felt that I had nothing to look forward to
18		I felt down-hearted and blue
19		I was unable to become enthusiastic about anything
20		I felt I wasn’t worth much as a person
21		I felt that life was meaningless
